# Plastic Surgery of the Face

**Published:** 1884-05

**Authors:** L. McLane Tiffany

**Affiliations:** Delegate from Medical and Chiurgical Faculty of Maryland, and Professor of Surgery in the University of Maryland.


AMERICAN JOURNAL

OF

DENTAL SCIENCE.

Vol. xviii. THIRD SERIES—MAY 1884. No. 1.

ARTICLE I.

### PLASTIC SURGERY OF THE FACE.

BY L. MCLANE TIFFANY, M. D.

(Delegate from Medical and Chiurgical Faculty of Maryland, and Professor of Surgery in the University of Maryland.

*Gentlemen*:—In the following remarks there will be no attempt made to present it in its entirety so large a subject as the one announced in the title, for to do so would require more time than the Association can spare and more wisdom than the writer can supply; the broad general principles which govern plastic facial surgery will be referred to, and then illustrated so far as may be proper by cases which have fallen under the writer's notice.

Plastic surgery of the face may well be, and usually is, divided into—

1st. Measures, for the restoration of parts congenitally at fault, either, from excess or deficiency.

2d. Measures for the restoration of parts deficient in consequence of accident or disease.

3d. Measures for the correction of deformity produced by cicatricial contraction; this last class being usually associated with the preceding, namely, an acquired deficiency of parts.

The first class of cases in which parts are congenitally wanting differs greatly from the two following:

1st. In that the deficiency results, during intra-uterine life, usually from failure of lateral halves to unite, and the deformity is apt to be near the middle line of the body.

2d. In that the edges and neighborhood of the deformity are usually well-nourished—normal tissue bounding the cleft.

3d. In that during early age the tissues are succulent and vigorous with life, thus lending themselves to new positions.

4th. In that the same deformity is seen again and again, marked hereditary predisposition being apparent sometimes, as in hare-lip and cleft palate, thus giving ample opportunity for study, and allows operator after operator to perfect old or devise new measures for relief, perfection so near as possible being gradually attained.

Far different, however, is it with the other classes. Here the deficiency occurs at any time and anywhere; the edges and vicinity of the affected part are made up of cicatricial tissue, and therefore are not well-nourished, do not stretch easily, and energetically refuse either to be transplanted or to afford nourishment to a superimposed flap. With the exception of *cancrum oris*, which occurs early in life, it is likely that no age is especially prone to accidental face deformity.

Still more is the comparison disastrous for acquired deformity when we reflect that practically never are similar lesions seen; the same type, possibly, but so very dissimilar in detail, for it may be said, with but scant fear of contradiction, that each case is to be studied by itself, and individual peculiarity must govern every operative measure; just as dwellings are built of brick and mortar, but into how many shapes are houses fashioned.

It would be well to recall the anatomical peculiarities of the region concerning which this paper treats, and how the face is built on an osseous framework of extremely irregular shape, containing cavities formed mainly by bones so thin that, being supplied as they are with periosteum on both surfaces, one face may be denuded, yet no necrosis follow, so free is the vascular supply, one periosteum being able to give nourishment to the entire thickness of bone—a physiological fact of very great importance, and extremely useful to the surgeon. The region overlaying the bones is the most mobile of the body, reflecting the ever active mental processes; the muscles are noticeable in that so many have attachment by one end to bone and by the other to muscle, principally the orbicularis oris, thus allowing great play of expression to the mouth, wherefore it is veiled in the sterner sex, as years multiply, by a lambrequin. The vascular supply is principally from the sides, by branches of the external carotid, and the motor nerve, portio dura of seventh, is from the same direction. Sensation is supplied by the fifth sending branches through the bones on which the soft parts rest. The great mobility already referred to, attained through the medium of connective tissue padding, extremely lax and very vascular, which renders this region peculiarly suited for plastic operations, for tissue carrying with it vascular channels after transfer, does not die, but grows well in its new situation, having nourishment sufficient.

Remedying a given deficiency comprises—

1st. Obtaining a piece of tissue to fill the hole and preparing its bed.

2d. Putting it in position and nourishing it.

3d. Filling the vacancy resulting from transfer.

4th. Ultimate result as regards usefulness and appearance.

To fill a hole in skin, of course skin must be taken, and it is to be chosen, as far as possible, similar to the lost piece. So far as possible, a transferred flap should be healthy; scar tissue must be rigidly excluded, and for this reason: that scar is nourished by subjacent parts, is not nourished laterally; if then it is raised and transferred, it sloughs, and no advantage follows.

It is well to call attention to the fact that support must be afforded to transplanted skin, especially about the mouth; and here the assistance of a competent dentist is to be invoked whenever necessary.

A flap should be cut, so that its long diameter corresponds with the known direction of the blood vessels, and with advantage an arterial branch may be included. The shape must depend upon the gap to be filled, and inasmuch as there is decided shrinkage when skin is raised from its bed, the flap should be much larger in all directions than the space which it is designed to fill. Such, however, is not always possible, and a flap may be transplanted partly to unite at once and partly by granulation. A flap that is to granulate will require usually a pedicle more broad than one which is to unite at once, since in the former case the process of granulation is to be supplied by the pedicle alone, while the latter, certainly at the periphery, is aided by the neighboring tissue. Incisions are to be made where possible in natural furrows and under cover—*e. g.*, under the chin, that the resulting scar may escape observation. I have derived great advantage from the homely custom of cutting a pattern of the deficiency, placing the pattern on sound tissue, and with ink or iodine marking out my flap. Enough subcutaneous tissue should be raised with the skin to insure a good vascular supply. Wolf's plan of skin flap without subcutaneous tissue or pedicle, is still on trial, and will doubtless prove of limited application. No muscle should be included. Bevelling the skin, as suggested by Packard, may be of use occasionally. The surface to which the flap is to be transferred must be prepared by freshening the edges and cutting away all cicatricial tissue, unless resting on a cushion of thick connective tissue, and even here, where there is a thick cushion, granulation will occur, and not immediate union. Of course if the flap edge has been bevelled the edge of skin against which it is to rest must correspond. Scar tissue, if adherent to bone, is to be removed, and a movable edge made beyond. The transplanted skin becomes adherent by gradulation, and in accordance with the opinion already expressed such a flap is to be fashioned with a broad base.

A certain amount of hemorrhage is always met with during, as well as after, the separation of the flap, the first incisions are especially bloody, but a most excellent hæmostatic is to be found in hot water, either poured over the wound or applied by pressing cloths, previously wrung out in it, against the oozing surface. The temperature should be about 120°, which will not be uncomfortable for the operator's hand. Hot water offers the additional advantage of keeping the flap warm and pliable. Especially are ligatures to be avoided, since they are a source of irritation and prevent early union; pressure and tortion will usually obviate the necessity for their employment. Where oozing is persistent I do not hesitate to lay a few strands of silver wire beneath the lower edge of the flap, to avoid retention of blood, and make firm with cotton wadding and a bandage, removing the wire after a certain number of hours, depending upon the hemorrhage, moderating compression at the same time.

Dissecting flaps from either side and sliding them to meet over a cavity is a certain way to insure failure; where the line of union is supported from beneath success is attained, not otherwise; even the upper lip congenitally fissured gives an unseemly result when so treated, yet here there is partial support.

The use of this procedure (sliding parts together) should be restricted to closing gaps left by transfer of skin to more distant parts; in other words, is complimentary to the major operation. In order to facilitate the sliding of flaps lateral incisions are occasionally of assistance, but only when ample blood supply is at hand and scaring may be disregarded, acombination of circumstances found more often upon the body where habitually covered by clothing than upon the face. Transfer of skin with a pedicle is more often available. This latter, the pedicle, should lie flat, and if transfer is made from a distance, the intervening skin is to be cut so as to make a bed for it to occupy, thus assisting immediate union and giving nourishment to the distal part of the flap. Exceptionally only may a pedicle bridge intervening tissue, as in the Taliacotian operation for a lost nose; for usually it, the pedicle, is taken from the border of the deformity. So far as possible, a pedicle should not be twisted, but it is often unavoidable; by forming the base of the flap judiciously, however, twisting is reduced to a minimum, and thus proper circulation ensured. If it so happen that a twisted pedicle remains elevated and unsightly, it can be pared and made to lie smoothly, but this only after the flap has become well settled in its new situation and vascular connection with surrounding parts established. Sensation after transfer skin gradually become normal, although immediately after operation it may be greatly perverted. The raw surface left by transfer of flap demands but scant courtesy; it is in healthy tissues, so that all sorts of liberties can be taken with it. Usually the edges are very freely dissected up, slided the one towards the other and united by suture, so as to form a linear scar; should tension be great, complete approximation is not attempted, and the intervening space allowed to granulate. Granulating surfaces I powder with iodoform and cover with absorbent cotton, a dressing which has given, in my hands, results more satisfactory than any other. Such a dressing may remain in place for a number of days; where a small surface is so covered, the removal of the first dressing may expose a well-formed scar.

If a flap fail to unite throughout, and healing by granulation be necessary, I use the same dressing. Raw surfaces are to be held together by suture: and of the various kinds I prefer the interrupted of silver wire, passed with a straight needle, not claiming for it, however, any special advantages over others. The pin suture so frequently used in plastic surgery I employ rarely. Whatever the suture used, or however used, one thing is essential: "there must be no tension on the lips of the wound;" for if there is, the stitches cut out, leaving a ragged wound, which heals slowly, with an irregular scar.

Tension then is to be avoided by free cutting, very free indeed, and by one or more deep sutures tied over quills or buttons. Such sutures are passed deeply at some distance from the wound and not showing it at all, and are drawn tightly enough to place the flap edge lightly in opposition with the proper surface previously denuded. There is then no tendency for the raw surfaces to separate, and union takes place rapidly. This is the great requisite to success, and it is easy to see of what small importance is the kind of suture which immediately unites the wound edges provided they already rest in apposition without tension. Cases sometimes present themselves where from one cause or another it is impossible to achieve the desired result at once. A series of operations are then to be done, each one gaining a little until success is brought about. The time intervening between successive operations depends upon the exigencies of the case, and follows no rule. Roux's well known case may be quoted, as reported in Velpeau, where seven operations were done, and which succeeded to the satisfaction of the operator in the end. The extension of scar tissue through the agency of multiple small incisions is too well known to require more than passing mention, and the same may be said of the necessity of giving rest to parts after operation by preservation of a suitable position.

It is scarcely necessary to state that a plastic operation, be it ever so simple, should be undertaken only when the patient is in excellent health; preparatory treatment is to be carried out by regulating digestion putting the skin in order by baths, exercise, etc., etc., for while it is true that but one part of the body is operated upon, yet all parts must contribute to a cure.

Inasmuch as it is my habit to precede tedious operations by a hypodermic injection of morphine, during preparatory treatment, I investigate the patient's susceptibility to the drug—especially is this necessary in children. Cool weather will always be more pleasant for both patient and operator than warm, and as plastic surgery is rarely a matter of momentary necessity, summer, certainly in town, should not be chosen as the time for operation.

Operations about the mouth, and particularly inside the month, present to the surgeon the problem how to prevent blood necessarily drawn from flowing into the trachea, and so asphyxiating the patient. Prior to the discovery of anæsthesia operations upon the mouth were undertaken with the patient seated, the head leaning somewhat forward, that the blood might escape through the lips; but as the erect position is imcompatible with ether or chloroform narcosis, an essential to operations at present, a provisional tracheotomy combined with some sort of pharyngeal occlusion is generally adopted. This permits the patient to keep the supine position during anæsthesia without fear of blood entering the air passages. But a tracheotomy is always a a complication to be avoided if possible, and so I have in a number of cases performed bloody operations of one kind or other about the mouth as follows: The patient receives a full dose of morphine hypodermically, and one-half hour later anæsthesia is induced (I have already referred, under the head of preparatory treatment to the necessity of finding out how morphine is borne by the patient) less than half an hour too much excitement. The patient is then turned face downwards, the thorax projecting beyond the edge of the table an assistant at each shoulder supports the upper parts of the body, and one standing behind the shoulder supports the head directing the face towards a window. The month is thus lower than the larynx and blood flows downwards and forwards from between the lips into a proper vessel, not back into trachea. I have removed from a patient in this position so much of the upper jaw as was bounded by the left central incisor and right posterior molar, opening both nostrils, yet no interruption from hemorrhage occurred. The surgeon is obliged to operate either kneeling or seated unless the table be of exceptional height.

